# CD200/CD200 receptor axis in psoriasis vulgaris

**DOI:** 10.1371/journal.pone.0230621

**Published:** 2020-03-23

**Authors:** Aisha A. Ismail, Hanaa M. Donia, Hafsa M. Ghatesh, Carmen I. Farid

**Affiliations:** 1 Department of Dermatology, Venereology and Andrology, Faculty of Medicine, Alexandria University, Alexandria, Egypt; 2 Department of Clinical and Chemical pathology, Faculty of Medicine, Alexandria University, Alexandria, Egypt; Medizinische Universitat Graz, AUSTRIA

## Abstract

**Background:**

Psoriasis is a chronic inflammatory multisystem disease with imbalance between the Th17 and T regulatory sub-populations. CD200/CD200R is an anti-inflammatory/immune-suppressive axis that might contribute to its pathogenesis given its relation to the Tregs induction. The current study aimed to investigate the status of the CD200/CD200R axis in the blood of psoriasis vulgaris patients versus healthy controls.

**Methods:**

In this comparative cross sectional study, the blood levels of sCD200 and CD200R levels were measured in 25 psoriasis vulgaris patients and an age and sex matched 25 healthy controls using ELISA and flow-cytometry respectively. Their levels were tested for correlation to disease severity.

**Results:**

sCD200 was significantly higher while CD200R was significantly lower in psoriasis vulgaris patients than in controls. They did not correlate to each other or to psoriasis severity although they differed significantly among cases of different severities.

**Conclusion:**

Aberrant expression of CD200/CD200R might play a role in psoriasis vulgaris pathophysiology and disease severity. It might constitute a future target of therapy, but cannot be used as a marker of disease severity.

## Introduction

Psoriasis is a chronic immune mediated disease of the skin with a growing list of multi-system comorbidities [1]. Inflammation, epidermal hyper proliferation and angiogenesis are the three key pathologic events taking place in psoriatic skin with inflammation being systemic rather than localized to the skin, which explains some of the comorbidities. [2,3].

Switching-off unneeded inflammation is the main function of Regulatory T cells (Tregs). They exert a suppressive action on several elements of both the innate and the adaptive immune systems through both direct and indirect mechanisms [4].

Their frequency in psoriatic blood and tissues varied among different reports; being either deficient [5], of normal frequency [6], or even of a higher frequency than in controls [7]. Regardless of their frequency, Tregs were found to be functionally defective in psoriasis in several reports. Isolated Tregs from the blood or tissues of psoriatic patients failed to suppress effector T cell responses consequent to their in-vitro stimulation, and several explanations have been suggested [8].

One of the main inducers of Tregs phenotype is the inhibitory CD200/CD200R signaling pathway. Both the receptor (CD200 R) and the ligand (CD200) are expressed on the surface of myeloid and some lymphoid cells. In addition, a soluble form of CD200 is found in the serum in two subtypes; a complete form (CD200s) acting as an agonist on the receptor, and an incomplete truncated form (CD200tr) acting as a competitive antagonist on the receptor [9].

Engagement of the ligand receptor couple leads to phosphorylation and activation of secondary signaling molecules mediating the inhibition of both MAPK (mitogen‑activated protein kinase) and NF‑κβ (Nuclear factor κβ). Consequently, the transcription and production of a myriad of inflammatory mediators is suppressed; of concern here are TNFα, IL‑1, and IL‑17; all of which are key mediators of inflammation in psoriasis [10]. This is paralleled by an increase in the synthesis of anti-inflammatory mediators; namely IL-10 and TGF-β. TGF‑β in particular is an inhibitory cytokine to both lymphocytes and macrophages and it polarizes the T cells towards differentiation into the Treg immune-phenotype (secreting more and more IL-10 and TGF-β) [9].

Moreover; agonist induced CD200R stimulation on macrophages leads to a selective inhibition of their IFN-γ and IL-17 induced cytokines and chemokines; hence it could be of value in switching off the auto-amplification loop of inflammation sustained by dendritic cells in psoriasis [11].

In activated lymphocytes; CD200/CD200R interaction leads to activation of the tryptophan catabolizing enzyme IDO (indoleamine 2, 3‑dioxygenase) producing the tryptophan metabolites kynurenines, which are strong apoptosis inducers in the CD4^+^ T cells [9]. Independent of its catabolic function, elevated IDO expression in dendritic cells promotes their conversion into tolerogenic phenotype that can induce and maintain the Tregs phenotype and suppressive action, while compromising other effector T cell proliferation and inducing their anergy. Tregs primed by MHCII molecule on the surface of pDCs in the absence of IDO lacked any suppressive activity [12].

Conversely, IDO expression in steady state pDCs is dependent upon interaction with Tregs. Hence there is a cross talk between pDCs and Tregs that is orchestrated by IDO and is necessary for the maintenance of self-tolerance in normal states and for switching off inflammation particularly during auto-inflammatory and autoimmune reactions [13].

The role of CD200/CD200R pathway has been investigated in several inflammatory diseases. Up regulation of CD200 with concomitant down regulation of its receptor (CD200R)—in peripheral blood or tissues- has been found in systemic lupus erythematosus (SLE) [14], inflammatory bowel disease [15] and rheumatoid arthritis[16], all of which–similar to psoriasis- are characterized by Th17/Treg cell imbalance [17]. sCD200 has been reported to be up regulated in psoriatic patients [18], but the condition of its receptor is currently unknown.

The current study aimed to investigate the status of this inhibitory pathway in psoriatic patients compared to healthy controls and its relation to disease severity.

## Materials and methods

The present comparative cross-sectional study included 25 non-arthritic psoriasis vulgaris patients recruited from the attendants of the Dermatology outpatient clinic of Alexandria Main University Hospital, Faculty of Medicine, Egypt, during the period from January 2016 to January 2018. They were compared to 25 healthy age and sex matched volunteers (by frequency matching) regarding serum soluble CD200 levels (measured by ELISA (Human CD200 PicoKine^™^ ELISA Kit, Boster Biological Technology, Pleasanton CA, USA, Catalog # EK0993), and CD200R expression level on both peripheral blood monocytes and lymphocytes (measured by flowcytometry using human CD200 R1 conjugated antibody(Human CD200 R1 Fluorescein-conjugated Antibody, Bio-Techne, Minneapolis, Minnesota, USA, Catalog # FAB3414F). Cells were analyzed on a FACSCalibur flow cytometer using CellQuest pro software (BD Biosciences, San Diego, CA, USA). Membrane fluorescence intensity was estimated and expressed as mean fluorescence intensity (MFI). Full laboratory protocol is provided by protocols.io: http://dx.doi.org/10.17504/protocols.io.6zihf4e. The used sample size gave an average calculated study power of 99% for the positive results.

Participants having any autoimmune or other inflammatory diseases, infections or malignancy were excluded. Patients who received any systemic medications or phototherapies during the previous three months or topical treatment for psoriasis within the previous month were also excluded. Alcoholics, and pregnant or breast feeding females were not included as well.

Disease severity in participating cases was assessed according to Psoriasis Area and Severity Index (PASI) score [19].

This study was approved by Alexandria faculty of medicine ethics committee and was carried out according to the principles of ethical human research in Helsinki declaration and its amendments. An informed written consent for enrollment in the study was obtained from all participants after discussing the study protocol.

Characteristics of the study population were recorded as frequency and mean or median according to normality of data distribution verified by The Kolmogorov- Smirnov tests. Chi-square test and Fisher’s Exact were used to test for association between categorical variables, Student t-test was used for normally distributed quantitative variables, while Mann Whitney test was used for abnormally distributed quantitative variables. Kruskal Wallis test was resorted to in case of testing association between multiple groups having abnormally distributed quantitative data and Post Hoc (Dunn's multiple comparisons test) for pairwise comparisons. Spearman coefficient was utilized to test for correlation between two abnormally distributed quantitative variables.

All statistical analyses were performed using IBM SPSS software package version 20.0. **(**Armonk, NY: IBM Corp**).** All *P*-values were two-tailed, and were considered significant when lower than 0·05.

## Results

In the current study, cases (n = 25) and controls (n = 25) did not differ significantly regarding their age or sex distribution (p = 0.992, P = 0.556 respectively). Most of the cases (72%) had early onset psoriasis (before 40 years), with a median disease duration of 8 years. All of the cases were of the vulgaris type of psoriasis. Median PASI score for the cases was 6.7 (range 0.60 to 39) with 60% of the cases having a mild disease (PASI<10). Table 1.

**Table 1:** Comparison between the two studied groups regarding studied clinical and biochemical data.

Characteristics	Cases (n = 25)	Controls (n = 25)	Test of significance
**Sex**			
Male (No. and %)	17 (68%)	15 (60%)	χ^2^ = 0.347, p = 0.556
Female (No. and %)	8 (32%)	10 (40%)	
**Age (years)**			
Min.–Max	17.0–60.0	18.0–60.0	t = 0.010, p = 0.992
Mean ± SD.	40.08 ± 14.42	40.12 ± 14.19	
**PASI score**			
Mild (<10)	15 (60%)	NA	
Moderate to Severe (≥10)	10 (40%)		
**CD 200 pg/ml**			
Min.–Max.	36.0–400.0	13.0–30.0	U = 0.0*^,^ p<0.001*
Median	70	17	power = 98.28%
**CD200R expression (lymphocytes) (MFI)**			
Min.–Max.	8.0–27.0	19.0–41.0	U = 11.50*, p <0.001*
Median	17	30 .0	power = 100%
**CD200R expression (monocytes) (MFI)**			
Min.–Max.	13.0–36.0	24.0–49.0	U = 31.50*^,^ p<0.001*
Median	23	37	power = 100%

χ^2^, p: χ^2^ and p values for **Chi square test** for comparing between the two groups

t, p: t and p values for **Student t-test** for comparing between the two groups

NA: not applicable

U, p: U and p values for **Mann Whitney test** for comparing between the two groups

*: Statistically significant at p ≤ 0.05

Differential WBC count was abnormal in 88% of our cases (22/25), of which neutrophilia was encountered in 5 cases (20%), neutropenia in 2 cases (8%), lymphocytopenia in 4 cases (16%), lymphocytosis in 2 cases (8%), monocytosis in 4 cases (16%), eosinophilia in 8 cases (32%), and basophilia in 3 cases (12%).

A highly significant higher level of sCD200 was found among cases as compared to controls (P<0.001), while a significantly lower level of CD200 R expression on both lymphocytes and monocytes was detectable in cases as compared to controls (P < 0.001 in both instances) Table 1.

Serum levels of sCD200 were significantly higher (p = 0.020) in patients having moderate to severe disease than in those with mild disease, but with no correlation to disease severity (p = 0.084). CD200R expression level on both lymphocytes and monocytes did not differ among groups of different disease severity and did not correlate with PASI score. Table 2.

**Table 2:** Relation and correlation between PASI score and different biochemical markers in cases group.

	PASI score		Test of significance	Correlation
	Mild (<10) (n = 15)	Moderate +Severe (≥10) (n = 10)		
**CD 200 pg/ml**				
Min.–Max.	36.0–110.0	58.0–400.0	**U =** 33.0*	**r**_**s =**_ 0.353, p = 0.084
Median	60.0	91.0	P = 0.020*	
			power = 73.84%	power = 42%
**CD 200R expression (monocytes)**				
Min.–Max.	13.0–31.0	15.0–36.0	**U** = 69.0	**r**_**s =**_ 0.259, p = 0.212
Median	22.0	24.0	P = 0.739	
			Power = 8.3%	power = 24%
**CD200R expression (lymphocytes)**				
Min.–Max.	10.0–27.0	8.0–24.0	**U** = 47.0	**r**_**s =**_ 0.362, p = 0.075
Median	14.70	17.50	P = 0.120	
			Power = 22.25%	power = 44%

U, p: U and p values for **Mann Whitney test** for comparing between the two groups

r_s_: Spearman coefficient

*: Statistically significant at p ≤ 0.05

No correlation was found between serum level of sCD200 and CD200R expression on either the lymphocytes or the monocytes in the group of cases (P = 0.633, P = 0.560 respectively).Table 3.

**Table 3:** Relation and correlation between CD200 and CD200R on monocytes and lymphocytes.

	CD200R expression Monocytes		Test of significance	Correlation
	Below median (≤23) (n = 14)	Above median (>23) (n = 11)		
**CD 200 pg/ml**				
Min.–Max.	36.0–400.0	38.0–360.0	**U =** 57.50	**r**_**s =**_ -0.123, p = 0.560
Median	80.0	60.0	P = 0.285	
			Power = 5%	power = 8.96%
	**(lymphocytes)**			
	**Below median (≤17) (n = 15)**	**Above median (>17) (n = 10)**		
**CD200 pg/ml**				
Min.–Max.	38.0–400.0	36.0–320.0	**U** = 57.0	**r**_**s =**_ -0.100, p = 0.633
Median	78.0	61.0	P = 0.318	
			Power = 13.7%	power = 7.59%

U, p: U and p values for **Mann Whitney test** for comparing between the two groups

r_s_: Spearman coefficient

*: Statistically significant at p ≤ 0.05

## Discussion

The significance of CD200/CD200R signaling pathway in several inflammatory and autoimmune diseases has been reported; its relation to the balance between the proinflammatory Th17 and the immuno-suppressive Tregs phenotypes has bee noted in several non dermatologic diseases[14–16]. However, very few publications are available on the role of this pathway in immune mediated skin diseases.

In the current study, we demonstrated a significantly higher level of sCD200 in the serum of psoriatic patients as compared to healthy controls. A similar observation has been made once by Akman-Karakaş A. et al. in a study that included 30 psoriasis vulgaris patients [18]. In the previously mentioned study, the authors did not mention excluding patients having comorbid arthritis or other systemic inflammatory conditions. The status of medication use was not mentioned either. Both comorbidities existence and medication use might influence the disease severity and the level of mediators at the time of assessment; hence their results needed to be verified bearing in mind these factors, which was done in our study.

Enhanced expression of sCD200 in psoriasis could be a consequence to the ongoing inflammatory response, characterized by an increased release of TNFα and INFγ, both of which are potent inducers of CD200 expression [9]. It could result also from the upregulation of P53 expression in psoriatic lesions [20]. CD200 gene is a target for p53 protein which is responsible for the upregulation of its product (CD200) in apoptotic cells in order to protect from an auto- immune reaction against exposed self-antigens [21].

Despite its up regulation and its documented inhibitory effects, it failed to control inflammatory activity in psoriasis as all our cases were presenting with an active disease phase. Moreover, its level was even significantly higher in patients with moderate to severe disease (according to PASI) than in those with mild disease. This could be explained by a possible up regulation of the truncated form of CD200 (CD200tr), which will act as an antagonist on the receptor leading to its inhibition rather than stimulation, and at the same time will lead to competition with the active soluble and membrane bound forms preventing their engagement with the receptor. Both the complete and truncated soluble forms of CD200 are measurable by the commercially available ELISA kits; hence we couldn’t differentiate whether the up regulation was due to overexpression of its agonistic or antagonistic forms. Factors leading to differential expression of the agonist or the antagonistic splice variant of sCD200 have not been reported yet.

An alternative explanation can be provided by our second finding which is a significant down regulation of the CD200R in our cases when compared to healthy controls. Membrane bound CD200 molecule lacks an intracellular signaling motif; hence its action is conveyed through its receptor [9]. Failure of CD200 to engage its receptor to elicit its inhibitory signal results in failure to induce its immune inhibitory action including polarization of T cells into Treg subset and inhibition of Th17 development; hence although the ligand is up regulated, it cannot induce its effect because of the deficient receptor expression. This dichotomy between CD200 and CD200R expression has been described in several other Th17 mediated diseases including SLE, inflammatory bowel disease and rheumatoid arthritis [14–16]. To the best of our knowledge, this is its first report in psoriasis vulgaris.

The receptor down regulation and consequent resistance to the inhibitory action of the axis might be an explanation in itself to the up regulation of the ligand (CD200), being an attempt of the body to overcome and control the persistent inflammation.

CD200R was down-regulated on both lymphocyte and monocyte populations, which indicates reduction of the inhibitory function of this pathway in both populations. In the myeloid lineage cells, CD200R down-regulation might result in perpetuation of their antigen presenting function (in this case self DNA/RNA complexed with LL37) and consequent stimulatory effect on naïve T cells polarizing them into the Th17 phenotype [11]. In the lymphocytes, deficient signaling through this inhibitory pathway might result in loss of its direct regulatory function on CD4 Tcells and down-regulation of the Tregs polarization along with its suppressive activity as is already observed in psoriatic patients. Both effects will aid in perpetuating the inflammation in psoriasis. This observation has been recently documented also in the skin of imiquimod induced psoriasis in mice, where CD200 R expression was found to be significantly lower in psoriatic mice skin when compared to normal controls [22].

CD200R expression regulation mechanisms are so far unreported. Increased expression on macrophages and microglia has been reported after their treatment by IL-4 and 13 in one study [23], while its level did not correlate to Th1(IL-2, IFN-γ) or Th2 (IL-4, IL-10) cytokine responses in another study. It did however correlate negatively with general serum markers of inflammation as ESR, and CRP levels in the latter study [24]. Both markers are found to be up-regulated in psoriasis which might be responsible for the down regulation of the receptor [25]. In rheumatoid arthritis patients, CD200R expression correlated negatively with the frequency of Th17 cells, and positively with the frequency of Tregs, indicating that it is the expression level of the receptor rather than the ligand that plays a significant role in the immune dysregulation characteristic of these diseases [24].

We speculated that the down regulation of the receptor might result from the over expression of its ligand as a counter balancing step to prevent possible excess immune-suppression or as a disease maintaining mechanism. We investigated if there was a relation between the expression levels of the ligand (sCD200) and its receptor (through induction or repression) as might happen in some other pairs of ligand/receptor interactions; e.g. glucocorticoid receptors being modulated by the ligand [26]. In our cases, there was no detectable correlation between the levels of the ligand and the receptor; hence the receptor expression is apparently independent of the concentration of its ligand despite the apparent inverse relation. This lack of correlation has not been verified in other studies involving measurements of CD200-CD200R expression levels so far. An experimental mouse model of psoriasis however reported up-regulation of CD200R upon subcutaneous injection of recombinant CD200, a change that was accompanied by symptom amelioration [22]. The contradiction in the findings might be explained again by the presence of multiple forms of CD200 in real life patients as in our cases with contradicting actions, all are measurable by ELISA, while in the experimental model, authors were able to observe the direct effect of the injected specifically prepared agonistic form of the ligand on its receptor. [22]

In the current study, although sCD200 serum level was significantly lower in patients with mild psoriasis than in those with moderate to severe disease, yet no correlation was detected between the expression of either the ligand (sCD200) or the receptor (CD200R) on either lymphocytes or monocytes and the severity of psoriasis expressed as PASI scoring. Akman-Karakaş et al. similarly reported lack of correlation between PASI and sCD200 in their studied psoriatic cases [18], but no available literature was found on the relation between severity and receptor expression. Regarding the status in other inflammatory Th17 mediated diseases, both CD200 and CD200R correlated negatively with rheumatoid arthritis severity (expressed as DAS28) [16] while they did not correlate with SLE severity scores [14].

Since CD200 over expression in psoriasis is likely mediated by higher levels of TNFα and INFγ [9], both of which contribute to the cytokine inflammatory storm in psoriasis, then difference in expression levels among different severities could be explained. However, the lack of correlation between disease severity and CD200 level is possibly due to the contribution of other several effector cytokines in the inflammatory process of psoriasis and consequently its clinical severity [10].

Determinants of CD200R expression on the other hand are less obvious; it did not correlate to any known Th1, Th2 or Th17 derived mediator in literature, which again explains lack of correlation to disease severity in our cases.

Both findings collectively indicate that CD200 or CD200R peripheral expression levels cannot serve as markers of disease severity in psoriasis vulgaris. They can be however exploited as targets for therapy in these patients and in other diseases characterized by Th17/Tregs imbalance given their balancing effect on these populations. Indeed, the use of infliximab and methotrexate in rheumatoid arthritis patients resulted in up regulation of both peripheral CD200 and CD200R, a change that correlated to the clinical improvement of the disease as evidenced by reduced disease severity scores. [16] In psoriasis, the subcutaneous injection of recombinant CD200 protein in imiquimod-induced psoriasis like inflammation in mice skin resulted in symptom attenuation, and reduction of serum levels of the pro-inflammatory mediators IL-6, IL-1β, and TNF-α. The results of this animal experimental model constitute direct evidence to the therapeutic utility of this axis in psoriasis.[22] Methods to modulate the agonistic ligand and the receptor should be the focus of oncoming research, and therapeutic trials utilizing possible modulators might reveal a valuable tool in the therapy of psoriasis.

## Conclusion

In conclusion, abnormalities in the expression of elements of the CD200/CD200R axis have been detected in psoriasis vulgaris patients in the form of peripheral overexpression of the ligand and down-regulation of the receptor when compared to normal controls. Given the immune suppressive and anti-inflammatory role of this pathway and its relation to Th17/ Tregs balance, they possibly play a role in the immune-pathogenesis of psoriasis, as reported in other inflammatory and auto immune diseases. Clinical trials utilizing modulators of this pathway should take part to investigate their possible utility in the management of psoriasis.

## Supporting information

S1 Fig (PNG)

S2 Fig (PNG)

S3 Fig (PNG)

S4 Fig (JPG)

S5 Fig (JPG)

S6 Fig (JPG)

S1 Table Relation between type of psoriasis according to age of onset and different parameters in cases group. (DOCX)

S2 Table Relation between family history, PASI score and biochemical markers in cases group. (DOCX)

S3 Table Relation between duration of disease (year), PASI score and biochemical markers in cases group. (DOCX)

S1 Data (XLSX)
