# Supplementary figures and images for: CD200/CD200 receptor axis in psoriasis vulgaris
Source: PLoS One. 2020 Mar 23;15(3):e0230621. doi: 10.1371/journal.pone.0230621 (PMC7089552; doi:10.1371/journal.pone.0230621)

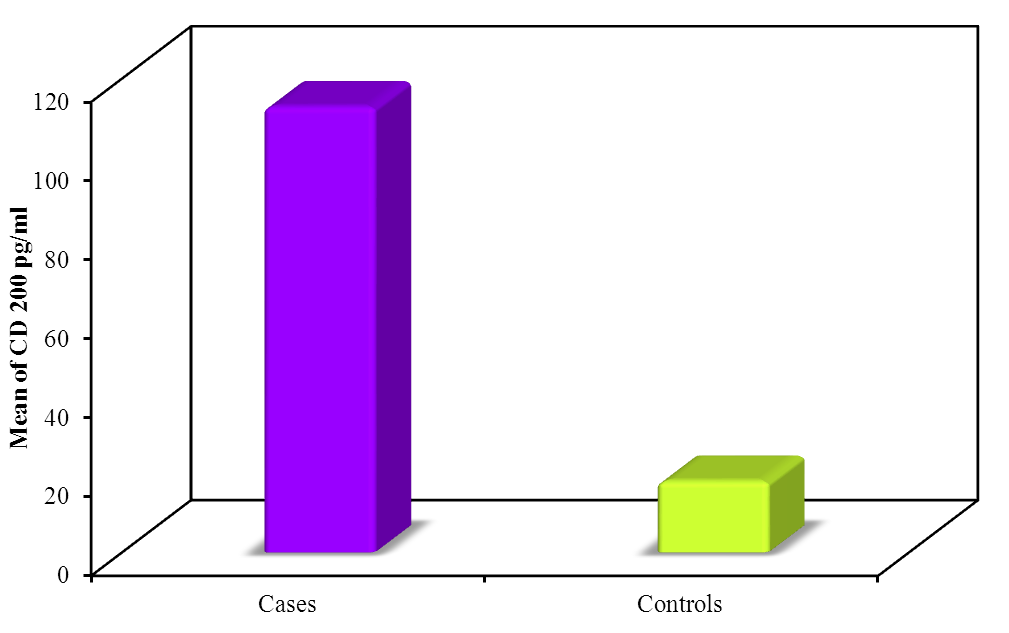

Supplement: S1 Fig — (PNG) [file pone.0230621.s001.png]

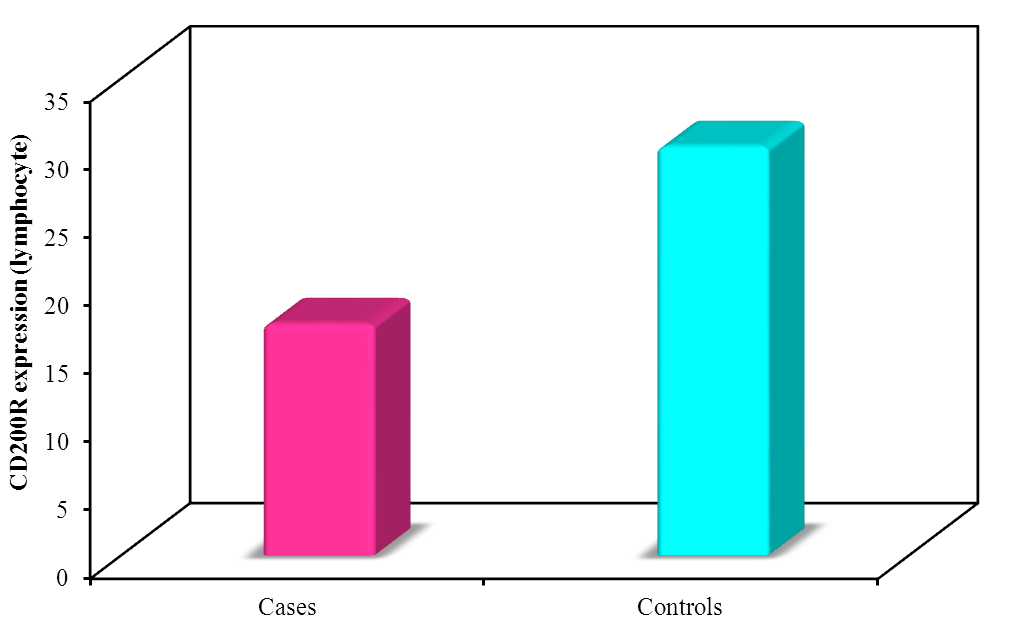

Supplement: S2 Fig — (PNG) [file pone.0230621.s002.png]

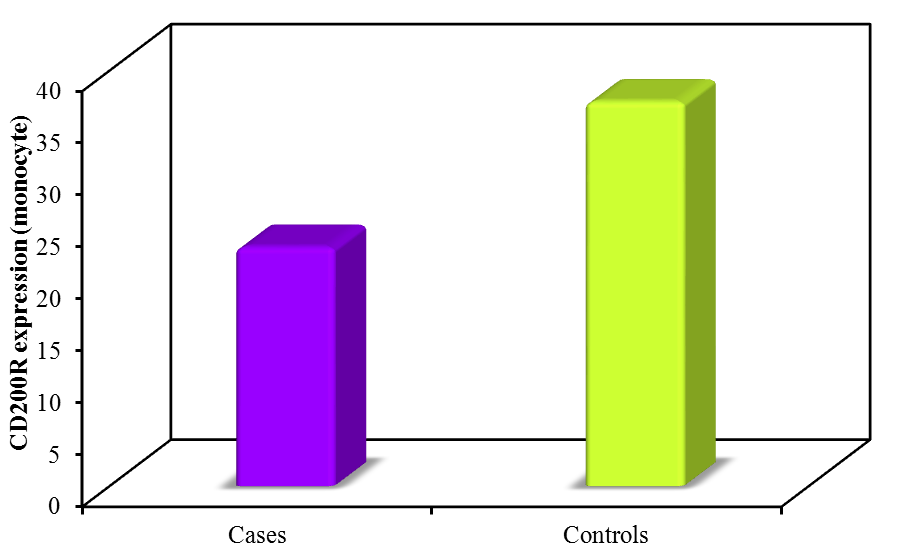

Supplement: S3 Fig — (PNG) [file pone.0230621.s003.png]

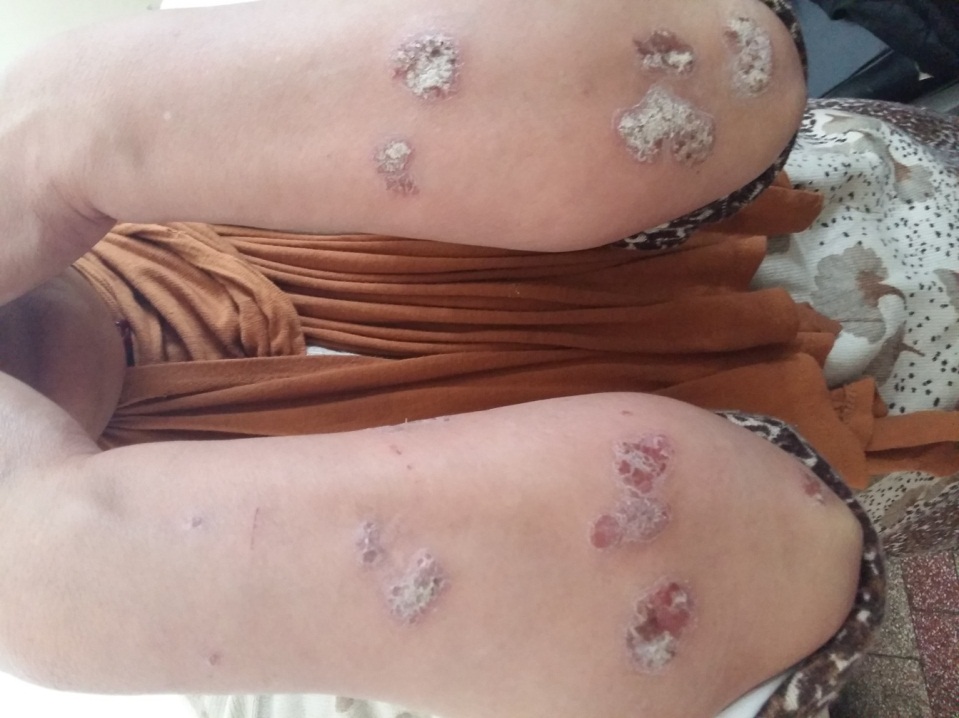

Supplement: S4 Fig — (JPG) [file pone.0230621.s004.jpg]

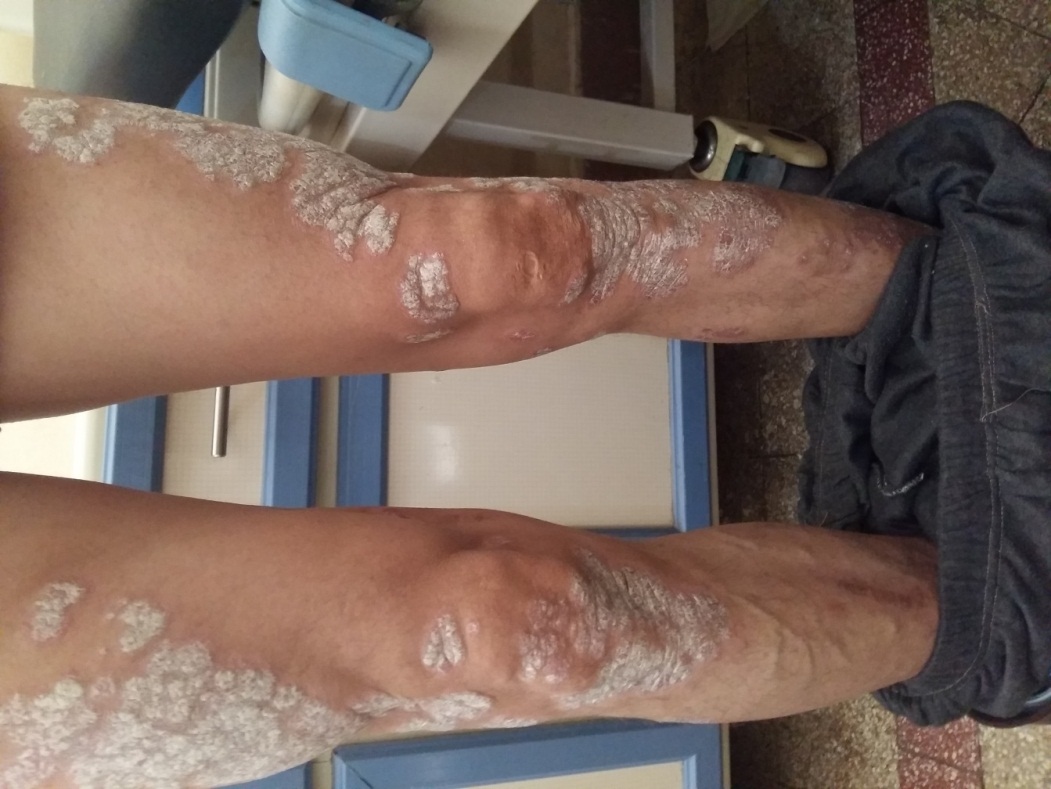

Supplement: S5 Fig — (JPG) [file pone.0230621.s005.jpg]

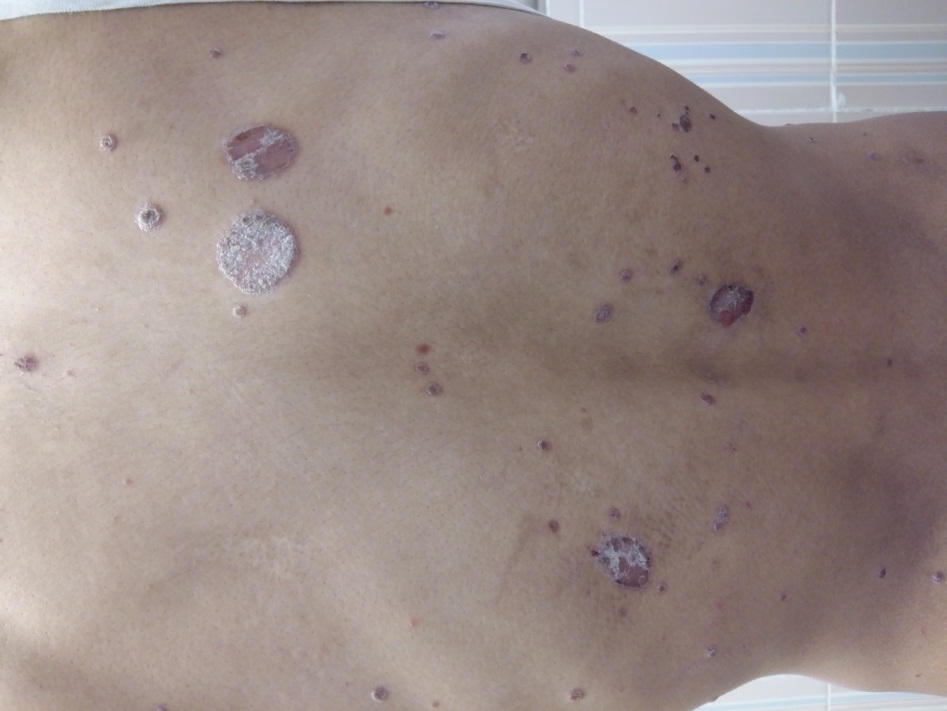

Supplement: S6 Fig — (JPG) [file pone.0230621.s006.jpg]
