# Supplementary material for: CD200/CD200 receptor axis in psoriasis vulgaris
Source: PLoS One. 2020 Mar 23;15(3):e0230621. doi: 10.1371/journal.pone.0230621 (PMC7089552; doi:10.1371/journal.pone.0230621)
Supplement: S1 Table — (DOCX) [file pone.0230621.s007.docx]

**Supplementary Table 1. Relation between type of psoriasis according to age of onset and different parameters in cases group**

|  | **Age of onset** | | | | **Test of sig.** | **p** |
| --- | --- | --- | --- | --- | --- | --- |
|  | **Psoriasis type1 (n= 18)** | | **Psoriasis type2 (n= 7)** | |  |  |
|  | **No.** | **%** | **No.** | **%** |  |  |
| **Family history** |  |  |  |  |  |  |
| No | 9 | 50.0 | 6 | 85.7 | χ^2^=3.217 | ^MC^p= 0.200 |
| First degree | 6 | 33.3 | 0 | 0.0 |  |  |
| Second-forth degree | 3 | 16.7 | 1 | 14.3 |  |  |
| **PASI score** |  |  |  |  |  |  |
| Mild (<10) | 11 | 61.1 | 4 | 57.1 | χ^2^=0.033 | ^FE^p= 1.000 |
| Moderate +Severe (≥10) | 7 | 38.9 | 3 | 42.9 |  |  |
| **CD 200 pg/ml** |  | |  | |  |  |
| Min. – Max. | 36.0 – 400.0 | | 52.0 – 89.0 | | U=56.0 | 0.672 |
| Median | 74.0 | | 62.0 | |  |  |
| **CD 200R expression (monocytes)** |  | |  | |  |  |
| Min. – Max. | 13.0 – 32.0 | | 17.0 – 36.0 | | U=62.0 | 0.952 |
| Median | 23 | | 21 | |  |  |
| **CD200R expression (lymphocytes)** |  | |  | |  |  |
| Min. – Max. | 8.0 – 24.0 | | 12.0 – 27.0 | | U=48.0 | 0.363 |
| Median | 15.80 | | 18 | |  |  |

χ^2^, χ^2^ and p values for **Chi square test** Sig. bet. grps was done using **Fisher Exact**

^MC^p: p value for **Monte Carlo** for Chi square test for comparing between the two groups

U, p: U and p values for **Mann Whitney test** for comparing between the two groups
