# Supplementary material for: CD200/CD200 receptor axis in psoriasis vulgaris
Source: PLoS One. 2020 Mar 23;15(3):e0230621. doi: 10.1371/journal.pone.0230621 (PMC7089552; doi:10.1371/journal.pone.0230621)
Supplement: S2 Table — (DOCX) [file pone.0230621.s008.docx]

**Supplementary Table 2. Relation between family history, PASI score and biochemical markers in cases group**

|  | **Family history** | | | | | | **Test of sig.** | **p** |
| --- | --- | --- | --- | --- | --- | --- | --- | --- |
|  | **No (n= 15)** | | **First degree (n= 6)** | | **Second degree (n= 4)** | |  |  |
|  | **No.** | **%** | **No.** | **%** | **No.** | **%** |  |  |
| **PASI score** |  |  |  |  |  |  |  |  |
| Mild (<10) | 10 | 66.7 | 2 | 33.3 | 3 | 75.0 | χ^2^=2.308 | ^MC^p= 0.442 |
| Moderate +Severe (≥10) | 5 | 33.3 | 4 | 66.7 | 1 | 25.0 |  |  |
| **CD 200 pg/ml** |  | |  | |  | |  |  |
| Min. – Max. | 36.0 – 360.0 | | 78.0 – 400.0 | | 52.0 – 82.0 | | H=9.157^*^ | 0.010^*^ |
| Median | 60.0 | | 215.0 | | 66.50 | |  |  |
| **Sig. bet. GRPS** | p_1_=0.005^*^,p_2_=0.483,p_3_=0.019^*^ | | | | | |  |  |
| **CD 200R expression (monocytes)** |  | |  | |  | |  |  |
| Min. – Max. | 16.0 – 36.0 | | 15.0 – 28.0 | | 13.0 – 25.0 | | H=0.725 | 0.696 |
| Median | 22.0 | | 23.0 | | 23.0 | |  |  |
| **CD200R expression (lymphocytes)** |  | |  | |  | |  |  |
| Min. – Max. | 10.0 – 27.0 | | 8.0 – 24.0 | | 11.0 – 21.0 | | H =0.167 | 0.920 |
| Median | 17.0 | | 15.85 | | 17.80 | |  |  |

χ^2^, χ^2^ and p values for **Chi square test** for comparing between the two groups

^MC^p: p value for **Monte Carlo** for Chi square test for comparing between the two groups

H, p: H and p values for **Kruskal Wallis test**, Sig. bet. grps was done using **Mann Whitney test**

p_1_: p value for comparing between no and first degree

p_2_: p value for comparing between no and second degree

p_3_: p value for comparing between first degree and second degree

*: Statistically significant at p ≤ 0.05
