# Supplementary material for: CD200/CD200 receptor axis in psoriasis vulgaris
Source: PLoS One. 2020 Mar 23;15(3):e0230621. doi: 10.1371/journal.pone.0230621 (PMC7089552; doi:10.1371/journal.pone.0230621)
Supplement: S3 Table — (DOCX) [file pone.0230621.s009.docx]

**Supplementary Table 3. Relation between duration of disease (year), PASI score and biochemical markers in cases group**

|  | **Duration of disease (year)** | | | | | | **Test of sig.** | **p** |
| --- | --- | --- | --- | --- | --- | --- | --- | --- |
|  | **First attack (n= 7)** | | **<10 (n= 9)** | | **≥10 (n= 9)** | |  |  |
|  | **No.** | **%** | **No.** | **%** | **No.** | **%** |  |  |
| **PASI score** |  |  |  |  |  |  |  |  |
| Mild (<10) | 7 | 100.0 | 5 | 55.6 | 3 | 33.3 | χ^2^=7.523 | ^MC^p= 0.036^*^ |
| Moderate +Severe (≥10) | 0 | 0.0 | 4 | 40.0 | 6 | 66.7 |  |  |
| **Sig. bet. GRPS** | ^FE^p_1_=0.088, ^FE^p_2_=0.011^*^,^FE^p_3_=0.637 | | | | | |  |  |
| **CD 200** |  | |  | |  | |  |  |
| Min. – Max. | 38.0 – 110.0 | | 45.0 – 400.0 | | 36.0 – 360.0 | | H=0.158 | 0.691 |
| Median | 80.0 | | 70.0 | | 62.0 | |  |  |
| **CD200R expression (monocytes)** |  | |  | |  | |  |  |
| Min. – Max. | 18.0 – 25.0 | | 13.0 – 36.0 | | 16.0 – 32.0 | | H=0.008 | 0.930 |
| Median | 20.0 | | 26.0 | | 24.0 | |  |  |
|  |  | |  | |  | |  |  |
| **CD200R expression (lymphocytes)** |  | |  | |  | |  |  |
| Min. – Max. | 10.0 – 27.0 | | 8.0 – 18.0 | | 16.0 – 24.0 | | H=8.753^*^ | 0.013^*^ |
| Mean±SD | 18.5±8.5 | | 13±5 | | 20± 4 | |  |  |
| **Sig. bet. GRPS** | p_1_=1.000, p_2_=0.039^*^,p_3_=0.004^*^ | | | | | |  |  |

χ^2^, χ^2^ and p values for **Chi square test** Sig. bet. grps was done using **Fisher Exact**

^MC^p: p value for **Monte Carlo** for Chi square test for comparing between the two groups

H, p: H and p values for **Kruskal Wallis test**

p_1_: p value for comparing between first attack and <10

p_2_: p value for comparing between first attack and ≥10

p_3_: p value for comparing between <10 and ≥10

*: Statistically significant at p ≤ 0.05
